# Increased risk of knee osteoarthritis progressing to total knee arthroplasty following patella fractures: an age stratified population analysis

**DOI:** 10.1007/s00590-026-04665-6

**Published:** 2026-02-02

**Authors:** Daniel E. Pereira, Zachary D. Randall, Mitchell S. Mologne, Mitchel R. Obey, Jenna-Leigh Wilson, Christopher M. McAndrew, Marschall B. Berkes

**Affiliations:** 1 Department of Orthopedics, Washington University in St. Louis, St Louis, USA; 2 Department of Orthopedics, University of California, Davis, Sacramento, USA

**Keywords:** Patella fracture, Post-traumatic arthritis, Total knee arthroplasty, Open reduction internal reduction, Orthopaedic trauma

## Abstract

**Introduction:**

Patella fractures are articular injuries that can alter knee biomechanics, disrupt joint contact forces, and promote cartilage degeneration. However, progression to reconstructive surgery is not fully characterized. This study investigates the risk of progression to total knee arthroplasty (TKA) following patella fractures and assesses whether initial operative versus nonoperative management impacts this risk.

**Methods:**

We retrospectively analyzed TKA progression in patients with patella fractures using synthetic data from a large Level I academic trauma center (1996–2024). Patients were identified by historic diagnostic codes for patella fractures and TKA. Age-stratified TKA rates were compared to published national data, and indirect standardization was used to calculate the age-adjusted standardized incidence ratio (SIR), risk difference (RD), and attributable risk percent (AR%).

**Results:**

Among 3212 native patella fractures, 263 patients (8.2%) later underwent TKA. The mean age at fracture was 58.1 years (SD 17.5) with a mean time to TKA of 4.1 years (SD 4.9). The SIR for TKA was 1.6 (95% CI 1.3–1.9), RD 3.1%, and AR% 37.2%. In the 406 operatively treated fractures (mean age 55.3 years, SD 18.6; 60.8% female), TKA occurred in 5.4% (SIR 1.4, 95% CI 0.9–2.2), compared to 8.6% in the 2,806 nonoperatively treated fractures (mean age 58.5 years, SD 17.4; 61.8% female; SIR 2.1, 95% CI 1.8–2.3) (*p* = 0.04).

**Conclusion:**

Individuals with patella fractures face an increased lifetime risk of advanced joint degeneration and subsequent TKA compared to the general population, with nonoperative treatment linked to a higher risk than operative management. Further analysis of initial injury patterns, radiographic findings, and patient factors are in need for further research to understand and validate these findings.

## Introduction

Patellar fractures are a relatively common intra-articular knee injury, with mechanisms that include direct impact to the anterior knee or over-tensioning of the extensor mechanism during eccentric contraction [1, 2]. Nonoperative management is typically indicated for patients in which the extensor mechanism is intact and with a fracture pattern demonstrating minimal displacement [3]. Surgical treatment typically involves open reduction and internal fixation (ORIF) with quadriceps or patellar tendon repair as needed, and is reserved for open fractures, significant displacement, or disruption of the extensor mechanism [3].

The patellofemoral joint is a cartilage rich compartment of the knee that contributes to the overall kinematics of knee motion. Accordingly, patellar fractures may lead to altered biomechanics, disordered joint contact forces, and cartilage degeneration [4]. To this point there is limited research into any increased risk of total knee arthroplasty (TKA) following a patella fracture, with a singular study reporting on an elevated risk of subsequent TKA after patella fractures [5]. Additionally, studies in arthroplasty have shown that patients with a history of a patellar fracture may be at an elevated risk of worse outcomes after routine TKA [6]. Specifically, patients who underwent TKA with a prior history of patella fractures are at a higher risk for periprosthetic joint infection, revision surgery, dislocation, and increased total cost [7].

The limited evidence in the literature indicates a possibly elevated risk of increased degeneration and elevated risk of progressing to arthroplasty surgery after patella fractures however this is still an area of needed research for validation and effect estimation. For that reason, this study investigates the risk of progression to TKA following patella fractures. The objectives of this study were to: (1) analyze the overall progression to TKA in patients with prior patellar fractures, (2) compare progression to TKA in patients who were treated operatively versus nonoperatively for their patellar fractures. We hypothesize that patients with a history of patella fractures may be at an increased risk of progressing to TKA compared to the general US population and that those treated operatively may have different cumulative incidence of future TKA compared to those treated nonoperatively.

## Methods

A retrospective cohort study was conducted to evaluate the incidence and risk of TKA following patella fractures treated either operatively or nonoperatively. Patients with historic diagnostic codes for patella fractures and subsequent TKA procedures were identified from January 1, 1996, to December 1, 2024, at a large Level I academic trauma center. Data were extracted from an institutional, de-identified synthetic data platform, MDClone (Beer Sheva, Israel), which is a validated internal database that generates encrypted data by using the distributions and characteristics of real patient data [8]. These datasets have been shown to be statistically comparable to the original patient data and is deemed IRB exempt at our institution [9–12].

Inclusion criteria for the study were: (1) treatment for a patella fracture, either operatively or nonoperatively, and (2) age above 18 years at the time of diagnosis. There was not a minimum time from fracture to follow-up, thus all patients were followed until December 1, 2024 for complete collation of all patients that fit inclusion criteria. Demographic data, including age, sex, and race, were recorded at the time of the fracture. Patients were categorized into six age groups: < 50, 50–59, 60–69, 70–79, 80–89, and 90 + years. The primary outcome was the incidence of TKA following patella fracture. TKA rates were calculated for each age group and compared to age-stratified national TKA rates obtained from published literature literature [13] to derive an age-adjusted standardized incidence ratio (SIR) via indirect standardization [14]. This method has been extensively used in medical literature including orthopedic studies [15–17]. Patients were further stratified based on their initial treatment type (operative vs. nonoperative), and TKA outcomes were analyzed separately for each group to identify treatment-specific risks.

Statistical analyses included t-tests for continuous variables and chi-square tests for categorical variables. The SIR of progressing to TKA was calculated with 95% confidence intervals. Risk differences (RD) and attributable risk percentages (AR%) were also computed. Time to TKA was analyzed as the mean number of years from fracture to surgery, with significance determined by two-tailed p-values. Results were presented as counts, percentages, means with standard deviations, and risk metrics. All analyses were conducted using R (version 4.3.3, R Foundation for Statistical Computing, Vienna, Austria), and *p*-values < 0.05 were considered statistically significant, significant values are labeled in result tables.

## Results

### Participant characteristics and initial treatment

A total of 3212 patella fractures were analyzed. Of these, 12.6% were operatively treated (n = 406) and 87.4% nonoperatively treated (n = 2806). (Table 1**.)** The operative group had a significantly lower mean age (55.3 vs. 58.5 years, *p* = 0.001). Gender distribution was similar between groups, with 60.8% female in the operative group and 61.8% in the nonoperative group (*p* = 0.76). Racial composition did not differ significantly, with 74.6% White in the operative group and 76.5% in the nonoperative group (*p* = 0.44). However, a higher proportion of censored race data was observed in the operative group (4.7% vs. 1.1%, *p* < 0.001).

**Table 1:** Baseline Characteristics and TKA Outcomes of Patients with Patella Fractures

Variable	Total	Operative	Nonoperative	*P*-value
N	3212	406 (12.6%)	2806 (87.4%)	NA
Age (SD) years	58.1 (17.6)	55.3 (18.6)	58.5 (17.4)	**0.001**
Female n (%)	1980 (61.6%)	247 (60.8%)	1733 (61.8%)	0.76
Male n (%)	1232 (38.4%)	159 (39.2%)	1073 (38.2%)	0.76
White n (%)	2450 (76.3%)	303 (74.6%)	2147 (76.5%)	0.44
Asian n (%)	38 (1.2%)	6 (1.5%)	32 (1.1%)	0.73
Black or African American n (%)	675 (21.0%)	78 (19.2%)	597 (21.3%)	0.37
Racial censored n (%)	49 (1.5%)	19 (4.7%)	30 (1.1%)	** < 0.001**
Percent with TKA	8.20%	5.40%	8.60%	**0.04**
Time to TKA (mean (SD), years)	4.1 (4.9)	1.23 (1.4)	4.16 (4.9)	**0.002**

*TKA* Total Knee Arthroplasty, *SD* standard deviation

### Risk of progressing to total knee arthroplasty after patella fracture

Of the 3,212 patella fractures, 263 patients (8.2%) underwent subsequent TKA. The mean time from fracture to TKA was 4.1 years (SD 4.9), with an average age at TKA of 66.5 years. Patients in the nonoperative group had a higher incidence of TKA compared to the operative group (8.6% vs. 5.4%, *p* = 0.04) and a significantly longer mean time to TKA (4.16 vs. 1.23 years, *p* = 0.002). The SIR of progressing to TKA was 1.6 (95% CI 1.3–1.9), the RD was 3.1%, and the AR% was 37.2%. Among operatively treated patients, the SIR of TKA was 1.4 (95% CI 0.9–2.2), while nonoperatively treated patients had an SIR of 2.1 (95% CI 1.8–2.3). A detailed age-stratified analysis of TKA incidence, categorized by initial operative versus nonoperative patella fracture management, is presented in Table 2.

**Table 2:** Age-Stratified Incidence of Total Knee Arthroplasty Following Patella Fracture

Age Group	Total Patella Fractures	TKA n (%)	Operatively Treated Fractures	TKA n (%) After Operatively Treated Fractures	Nonoperatively Treated Fractures	TKA n (%) After Nonoperatively Treated Fractures	P-value
< 50	583	21 (3.6%)	122	7 (5.7%)	461	14 (3.0%)	0.17
50–59	397	32 (8.1%)	50	5 (10.0%)	347	27 (7.8%)	0.58
60–69	694	56 (8.1%)	85	4 (4.7%)	609	52 (8.5%)	0.22
70–79	811	92 (11.3%)	92	6 (6.5%)	719	86 (12.0%)	0.12
80–89	547	58 (10.6%)	47	0 (0.0%)	500	58 (11.6%)	** < 0.01**
90 +	180	4 (2.2%)	10	0 (0.0%)	170	4 (2.4%)	1.0
Total	3212	263 (8.2%)	406	22 (5.4%)	2806	241 (8.6%)	0.04

*TKA* Total Knee Arthroplasty

## Discussion

This study evaluates the risk of TKA following patella fractures, stratified by operative versus nonoperative fracture treatment. Our findings demonstrate that 8.2% of patients with prior patella fractures eventually underwent TKA, with a significantly higher incidence in the nonoperative group (8.6%) compared to the operative group (5.4%, *p* = 0.04). The mean time from fracture to TKA was 4.1 years, and the SIR of progressing to TKA was 1.6 (95% CI: 1.3–1.9), showing an elevated risk of TKA incidence compared to the general population. Additionally, patients treated nonoperatively not only had a higher incidence of TKA but also had a significantly longer time to TKA compared to their operatively treated counterparts (4.16 vs. 1.23 years, *p* = 0.002). These findings contribute to the limited literature assessing long-term knee degeneration following patella fractures and highlight the potential impact of initial treatment choice on future joint health.

Patients who experienced patellar fractures were at a significantly higher risk of TKA as compared to the general population. This is in alignment to prior studies within the literature, which have shown that periarticular and intraarticular injury increase the risk for later TKA [18]. This is attributed, in part, from subsequently abnormal biomechanics, cartilage damage from the initial injury, and altered joint contact forces leading to asymmetric and incongruous cartilage loading stress. Additionally, it is theorized that a large contributing factor to degenerative changes in the knee following patellar fracture may occur as a result atrophy of the quadriceps muscle [19]. In a cohort of 30 patients, Lazaro reported that patients with patella fractures had significant deficits in knee extensor strength, power, and endurance at 12 months post-operative as compared to their unaffected knee [19]. A suboptimal extensor mechanism potentially attenuates the action of the quadriceps, thus requiring a higher force generated by the quadriceps [20]. Quadriceps weakness has been associated with osteoarthritis and is theorized to have a contributing factor in the pathogenesis of the condition [21, 22]. An atrophic quadriceps muscle may also lead to patellar maltracking, which is further complicated by tightness in surrounding structures such as the lateral retinaculum that may have been damaged in the initial injury or during the surgical procedure [19, 23]. Another contributing factor may be the loss in range of motion, as LeBrun and colleagues reported that 20% of their cohort of patellar fractures experienced an extensor lag of greater than 5 degrees as well as 38% of patients experiencing a restriction in flexion over 5 degrees [24].

Patients who were treated non-operatively were at a significantly higher risk of later TKA than patients who were treated surgically. This is likely multifactorial in nature. First, while many of these patients treated nonoperatively may have an intact extensor mechanism, it may be compromised in terms of vector of force, strength, and stamina. Specifically, while indications for surgery include displacement of the articular surface or extensor mechanism compromise there may be multiple reasons for why nonoperative management was chosen; such as patient related comorbidities. Furthermore, in those where appropriate nonoperative management was chosen, there may be sub-clinical development of malunion or intra-articular displacement, which may develop over time and may not be seen by clinicians during routine follow up [25]. This could lead to incongruous contact area between the patella and femur, leading to nonanatomic and asymmetric forces on the articular surfaces of the joint [20, 26]. This in turn may cause degenerative changes to both the articular cartilage and underlying bone. Indeed, articular step-off of greater than 2 mm has been associated with increased risk of post-traumatic arthritis in some studies however the extent of impact and generalizability across injured joints is uncertain [27–29]. Moreso, true step-off distances may be underestimated, as even anatomic reductions of patellar fractures assessed by radiographs showed articular cartilage step-off in over 11% of patients when later assessed arthroscopically 12 months later [25]. In contrast, while surgical intervention carries inherent risks, it should achieve improved anatomic realignment, re-establish the extensor mechanism, and reconstitutes more optimal biomechanics of the patellar apparatus. In addition, it allows early and more aggressive rehabilitation which may prevent further quadriceps atrophy and promote protective stabilization of surrounding ligamentous and muscular structures. However, patients who underwent surgical intervention may also be less likely to proceed with subsequent TKA due to reluctance to undergo another operation after their patella ORIF. This hesitation, combined with improved anatomic alignment, extensor mechanism restoration, and the benefits of early rehabilitation, may contribute to the lower conversion rates in this population. Lastly, while lower in rate, operatively treated patella fractures received TKA at less time from injury than nonoperatively treated. This possibly elucidates a minority population within operatively treated patella fractures that developed post-traumatic degeneration at a greater severity or rate than the combined cohort. However, it may also be a consequence of the higher energy placed on the knees of operatively treated patella fractures. Accordingly, further studies should seek to understand this rapid degeneration following fixation.

There are important limitations to consider for this study. While the reliability and reproducibility of synthetic databases is nascent, it has been shown to be equivalent to referencing real patient information. However, there are limitations to this tool such as the amount of censorship of the data. While low in our database, it may limit generalizability. Another limitation was the lack of minimum time from fracture to follow-up; however, we feel this was clinically appropriate as many patients experienced progression to TKA within 1 year of fracture. Additionally, as increased variables are included in the databases, such as patient comorbidities, so does censorship, further limiting comparison. For that reason, this study sought to analyze population level trends with the aim of promoting further study and elucidating broad associations. This study’s retrospective nature and single institution source may limit its’ generalizability. However, this study is one of the first to present population level associations between patella fractures and joint degeneration as well as the potential protective nature of surgical fixation in its’ natural history. This study should animate further, multi-institutional study on the natural history of nonoperatively treated patella fractures.

## Conclusion

Patients with patella fractures face an increased lifetime risk of advanced joint degeneration and subsequent TKA compared to the general population, with nonoperative treatment linked to a higher risk than operative management. Limitations precluded analysis of initial injury and patient factors, highlighting the need for further research to understand and validate these findings. Further research is needed to assess the benefit of surgical intervention on patella fractures including the impact of anatomic reduction, fixation, and early mobilization on preventing advanced joint degeneration.

## Data Availability

No datasets were generated or analysed during the current study.
